# Neutrophils: New Critical Regulators of Glioma

**DOI:** 10.3389/fimmu.2022.927233

**Published:** 2022-07-04

**Authors:** Guanyu Wang, Jinpeng Wang, Chaoshi Niu, Yan Zhao, Pengfei Wu

**Affiliations:** ^1^ Department of Neurosurgery, The Second Affiliated Hospital of Harbin Medical University, Harbin, China; ^2^ Department of Urology, The Second Affiliated Hospital of Harbin Medical University, Harbin, China; ^3^ Department of Neurosurgery, The First Affiliated Hospital of USTC, Division of Life Sciences and Medicine, University of Science and Technology of China, Hefei, China; ^4^ Anhui Provincial Stereotactic Neurosurgical Institute, Hefei, China; ^5^ Anhui Province Key Laboratory of Brain Function and Brain Disease, Hefei, China; ^6^ Anhui Provincial Clinical Research Center for Neurosurgical Disease, Hefei, China; ^7^ Anhui Province Key Laboratory of Translational Cancer Research, Bengbu Medical College, Bengbu, China

**Keywords:** glioma, neutrophils, tumour microenvironment, immunosuppression, treatment

## Abstract

In cancer, neutrophils are an important part of the tumour microenvironment (TME). Previous studies have shown that circulating and infiltrating neutrophils are associated with malignant progression and immunosuppression in gliomas. However, recent studies have shown that neutrophils have an antitumour effect. In this review, we focus on the functional roles of neutrophils in the circulation and tumour sites in patients with glioma. The mechanisms of neutrophil recruitment, immunosuppression and the differentiation of neutrophils are discussed. Finally, the potential of neutrophils as clinical biomarkers and therapeutic targets is highlighted. This review can help us gain a deeper and systematic understanding of the role of neutrophils, and provide new insights for treatment in gliomas.

## Introduction

Gliomas are heterogeneous and primary malignant tumour in the brain. Glioblastoma (GBM) is the most lethal form of glioma, accounting for 70-75% of all diagnoses of diffuse glioma and having a median overall survival (OS) time of 14-17 months (1). The current standard of treatment includes maximal surgical resection and combined radiochemotherapy (2, 3). The significance of Stupp protocol has become the standard of care for the treatment of GBM. It consists of radiotherapy and concomitant chemotherapy with temozolomide, an alkylating agent (4). Over the years, many arts have been improved to aid the surgeon in the resection about the brain cancer. Improvements such as surgical microscopes, high-​resolution imaging, fluorescence-​guided surgery and neuronavigation are widely used in glioma treatment (5–7). Despite aggressive treatment strategies over the past few decades, the OS of glioma patients has not improved significantly due to the rapid proliferation, extensive invasion, and treatment resistance of gliomas (8). GBM tumours are highly resistant to treatment and the resistance can be explained by characteristics of TME (9). The GBM microenvironment contains many different non-cancerous cell types in addition to cancer cells, including endothelial cells, pericytes, fibroblasts and immune cells. These cells interact with one another and with tumour cells to perpetuate brain tumour growth (10). A state of immunosuppression characterizes GBM’s TME, thanks to the secretion of several cytokines by tumour cells, microglia, and tumour associated macrophages (TAMs) (11). In contrast to other immune cells, comparatively less is known about the contributions of neutrophils.

Neutrophils play various roles in different diseases. Neutrophils exert antimicrobial and inflammatory functions through phagocytosis, degranulation, release of neutrophil extracellular traps (NETs) and antigen presentation (12, 13). Neutrophils release decondensed DNA fibres and antimicrobial peptides, known as NETs (14). These web-like structures trap and kill different bacteria (14), fungi (15, 16), and parasites (17). At present, the importance and role of neutrophils in cancer have increased over the past decades (18). And neutrophils play an oncogenic role primarily by increasing DNA damage, angiogenesis and immunosuppression (19). The association between tumour initiation and progression, cancer-associated thrombosis and NETs has been reported (20–24).

Increasing evidence reveals that the numbers of circulating and tumour-infiltrating neutrophils are relevant to immunosuppression, poor survival and a poor prognosis in patients with cancer (25–27). However, the role of neutrophils in cancer is a controversial issue. The results of many studies have shown that tumour-associated neutrophils (TANs) are able to stimulate tumour cell migration and invasion (28–30). Conversely, findings from many other studies have suggested that TANs have various antitumour properties, such as direct cytotoxicity against tumour cells and inhibition of metastasis (31–33). Additionally, neutrophil classification in the TME, such as N1/N2 neutrophils and polymorphonuclear myeloid-derived suppressor cells (PMN-MDSCs), is also a controversial topic (34, 35). In gliomas, Using immuno-histochemical analysis of glioma sections, Fossati G et al. reported that neutrophil infiltration into tumours was significantly correlated with glioma grade (36). Subsequently, researchers found that increased neutrophil recruitment during antiangiogenic therapy promoted glioma progression and might promote treatment resistance (37). In addition, studies have found that the neutrophil-to-lymphocyte ratio (NLR) of patients with glioma is valuable for prognosis and diagnosis (38). We reviewed the recent association of neutrophils with gliomas and found that gliomas are characterized by an immunosuppressive TME. Pathologically activated neutrophils, called PMN-MDSCs, are a type of myeloid-derived suppressor cell (MDSC) and one of the major contributors to the immunosuppressive properties of gliomas (39, 40). As a consequence, neutrophils are now the subject of intense research in gliomas.

However, our understanding of the roles of neutrophils in gliomas is still limited to date. This article aims to review neutrophil research in cancer patients. The search was focused on the association of circulating neutrophils and tumour-infiltrating neutrophils with prognosis in glioma patients. A PubMed search using the keywords “neutrophils”, “gliomas”, “tumour microenvironment”, “myeloid-derived suppressor cells” and “neutrophil-to-lymphocyte ratio” was performed. Reference lists were then searched for additional articles. Available data were obtained from patients with glioma to elucidate the roles of neutrophils with various phenotypes in gliomas. In addition, we dissected the pathways that mediate the transport of neutrophils to the tumour site, described their role once they arrived in the tumour microenvironment, and integrated this with the current understanding of glioma progression. A vast body of evidence supports the importance of the neutrophils in the progression of gliomas, and the possibility of neutrophils in the treatment of glioma is further discussed in this paper in combination with recent studies. Therefore, elucidating the mechanisms by which glioma cells interact with neutrophils can uncover multiple potential therapeutic targets for clinical applications.

## Characteristics of Neutrophils in Cancer

Neutrophils are derived from the bone marrow and give rise to multiple granulocytic immune cell subsets (41). In a steady state, normal adults produce more than 1×10^11^ neutrophils per day (42). Neutrophils have long been considered as cells playing a crucial role in the immune system. They participate in the inflammatory response in the body and are the first line of defense against pathogen invasion (12). Inflammation responds to infection and carries out wound healing and tissue regeneration. Inflammation plays an important role in protecting the body. However, chronic inflammation induces cancer by destroying tissues. For example, chronic hepatitis increases the risk of liver cancer (43). Neutrophils provide a link between inflammation and cancer.

In recent years, researchers found neutrophils within tumours in the majority of solid tumour samples (44). Several studies have revealed a correlation between the presence of neutrophils and a poor prognosis in patients with early-stage melanoma, head and neck cancer or hepatocellular carcinoma and demonstrated that the presence of neutrophils is independently associated with a poor prognosis (45–47). In an in-depth study of neutrophils, it was found that neutrophils are an important component of the TME (48). In the TME, neutrophils have varied functions and have been classified using different terms, including N1/N2 neutrophils, TANs, and PMN-MDSCs (49–51). In 2009, Fridlender et al. classified the types of antitumorigenic and protumorigenic TANs, named N1 and N2, respectively. They showed that transforming growth factor-β (TGF-β), an immunosuppressive cytokine overexpressed by tumour cells, polarized neutrophils into a protumorigenic phenotype (N2) and that neutrophil depletion caused a small decrease in tumour growth in mouse models. However, the presence of interferon β (IFN-β) or blockade of TGF-β with SM16, an oral inhibitor of TGF–β receptor kinase, led to the aggregation of neutrophils with an antitumorigenic phenotype (N1) (52, 53). In this case, TANs depletion led to increased tumour growth (35, 54). Despite the existence of functional differences, no definitive surface markers have been identified to distinguish N1 and N2 TANs (35). Although there is no obvious surface marker of N1/N2 at present, The classification of N1 and N2 used to refer to antitumour and protumour neutrophils is important for our understanding of the role of neutrophils in tumours. We hope that interested readers can conduct follow-up studies to distinguish the N1/N2 classification of neutrophils.

TANs have important roles in cancer initiation and progression, and high densities of neutrophils are correlated with more advanced-stage disease in patients with gastric cancer and are more likely to be detected in more aggressive pancreatic tumours (55, 56). Several studies involving patients with early-stage melanoma, head and neck cancer, and hepatocellular carcinoma have revealed a correlation between presence of TANs and a poor prognosis (45–47). However, other papers of mouse metastatic renal cell carcinoma models have highlighted the antitumour potential of neutrophils. The antitumour neutrophils recruited to the lung by tumour-secreted chemokines build an antimetastatic barrier (54, 57). Hepatocyte growth factor/MET proto-oncogene-dependent nitric oxide release by antitumour neutrophils promotes cytotoxicity, which abates mouse Murine Lewis lung carcinoma cells, melanoma cells and human non-small-cell lung carcinoma cells growth and metastasis (58). Interestingly, in the colorectal cancer (CRC), the prognostic relevance of TANs is controversial. Rao H.L et al. discovered that the presence of CD66b^+^ neutrophils detected in 229 CRC patients using tissue microarray and immunohistochemistry. And neutrophils were identified as an independent factor for a poor prognosis in patients with CRC (59). In contrast, data from early stages of colon cancers patients have suggested that infiltration of CD66b^+^ neutrophils in the tumour front is associated with a favourable prognosis in patients with colon cancers (60). The differences in the conclusions of these studies may differ from the selected study patients, which included only colon cancers and not rectal cancers in the second study. In addition, manual counting of neutrophils according to their morphology may influence the results. The role of neutrophils in lung cancer is also controversial. In a study involving patients with early-stage (stage I–III) non-small cell lung carcinoma (NSCLC), high CD66b^+^ neutrophil density had a significantly effect on increased relapse following surgical resection and had a trend toward decreased OS (61). The presence of CD66b^+^ TANs show diverging prognostic effect in NSCLC patients according to histological subgroups. CD66b^+^ TANs described as a positive prognostic factor in patients with squamous cell carcinoma but an adverse prognostic factor in those with adenocarcinoma (62). Since there is no consensus on methods for staining and identifying neutrophils in cancer tissues, the prognostic implications of neutrophil infiltration in these patients clearly require further investigation.

Apart from the TANs, when describing the role and importance of neutrophils in cancer, PMN-MDSCs cannot be ignored. In 2007, MDSCs were confirmed and defined with this canonical name. MDSCs are a heterogeneous population of immature myeloid cells with immunosuppressive functions. Granulocytic or polymorphonuclear MDSCs (G/PMN-MDSCs), early-stage MDSCs (eMDSCs) and monocytic MDSCs (M-MDSCs) are the main types of MDSCs that have been detected (34, 63). Based on the typical suppressive functional characteristics of MDSCs, it has been suggested that PMN-MDSCs are a population of neutrophils with immunosuppressive activity (29, 64, 65). MDSC production follows the same differentiation pathway as the production of neutrophils and monocytes, both of which are produced by granulocyte colony-stimulating factor (G-CSF) and macrophage colony-stimulating factor (M-CSF) stimulation (39, 66, 67). Accumulating evidence indicates that the ability to suppress T cells is an important characteristic of MDSCs. The potent immunosuppressive activity of MDSCs is the reason that the function of MDSCs is different from that of monocytes and neutrophils. In addition, mature neutrophils (CD14^-^ CD15^+^ CD66b^+^ CD16^+^) express specific cell-surface proteins (68). In mice, SiglecF^high^ CD11b^+^ Ly-6G^+^ Gr-1^+^ cells resemble neutrophils (69). MDSCs are generally characterized as expressing the myeloid lineage differentiation antigen Gr-1 (Ly6G and C); CD11b, M-MDSCs typically have the phenotype CD11b^+^ Ly6C^high^ Ly6G^−^; and PMN-MDSCs are typically defined as CD11b^+^ Ly6C^low^ Ly6G^+^ (70). A more complex panel of markers is typically used to identify human MDSCs (CD11b, CD14, CD15, CD66b, HLA-DR and CD33), M-MDSCs (CD14^+^ CD15^-^ HLA-DR^−/low^) and PMN-MDSCs (CD14^-^ CD15^+^ CD66b^+^ CD16^+^ CD11b^+^ CD33^+^ HLA-DR^-^) (71–73) (The markers summarized in **Table 1**). These markers have also been shown to be expressed by neutrophils. Therefore, we have concluded that the term PMN-MDSCs actually describes a subset of neutrophils until more definitive evidence is found.

**Table 1:** Markers of neutrophils and MDSCs.

	Neutrophils	MDSCs	G/PMN-MDSCs	M-MDSCs
Human	CD14^-^ CD15^+^ CD66b^+^ CD16^+^	CD11b CD14 CD15 CD66b HLA-DR CD33	CD14^-^ CD15^+^ CD66b^+^ CD16^+^ CD11b^+^ CD33^+^ HLA-DR^-^	CD14^+^ CD15^-^ HLA-DR^-/low^ CD33
Mice	CD11b^+^ Siglec-F^–^ Gr-1^+^ Ly6G^high^	CD11b Gr-1	CD11b^+^ Ly6G^+^ Ly6C^low^	CD11b^+^ Ly-6G^-^ Ly6C^high^

## Functional Roles of the NLR and Circulating Neutrophils in Glioma

Circulating neutrophils are non-negligible component of the inflammation, which plays important roles in cancer development and progression (74). The NLR, a systemic cellular inflammation marker, is a noninvasive biomarker for patients with cancer. We calculated the NLR as follows: NLR = neutrophil count/lymphocyte count (75). The NLR is a low-cost method, as lymphocyte and neutrophil counts can be easily derived using the common complete blood count (76). The NLR has become a prognostic indicator for survival in many tumour types, including CRC, hepatocellular carcinoma, breast cancer and gliomas (38, 77–79).

Concerning gliomas, the NLR is a widely used parameter for diagnosis and OS prediction (80, 81). The approach has shown diagnostic value in differentiating isocitrate dehydrogenase-mutant (IDH-mt) GBM from IDH-wild-type (IDH-wt) GBM (82). Auezova et al. found lower NLR values in patients with IDH-mt GBM (83). A systematic review found that high NLR values were associated with lower overall survival and that patients with a high NLR value were associated with high-grade gliomas (38). In addition, a retrospective review reported that a lower NLR was associated with longer OS during focal radiotherapy and concomitant temozolomide treatment (84). However, NLR can potentially be affected by bacterial or viral infections or drug treatments (85). For example, bacterial infections and steroid usage can increase neutrophil counts, while viral infections may increase lymphocyte counts. The effects of acute disease conditions on NLR may overlap with chronic persistent inflammation. In addition, hypertension, diabetes mellitus, metabolic syndrome, left ventricular dysfunction and hypertrophy, acute coronary syndromes, cardiovascular diseases, abnormal thyroid function tests, renal or hepatic dysfunction, previous history of infection (<3 months), inflammatory diseases, and some medications (e.g. steroids) can potentially affect the measurement of NLR (76). Therefore, the measurement of NLR should consider the potential effects of other conditions or drug use.

**Figure 1:**
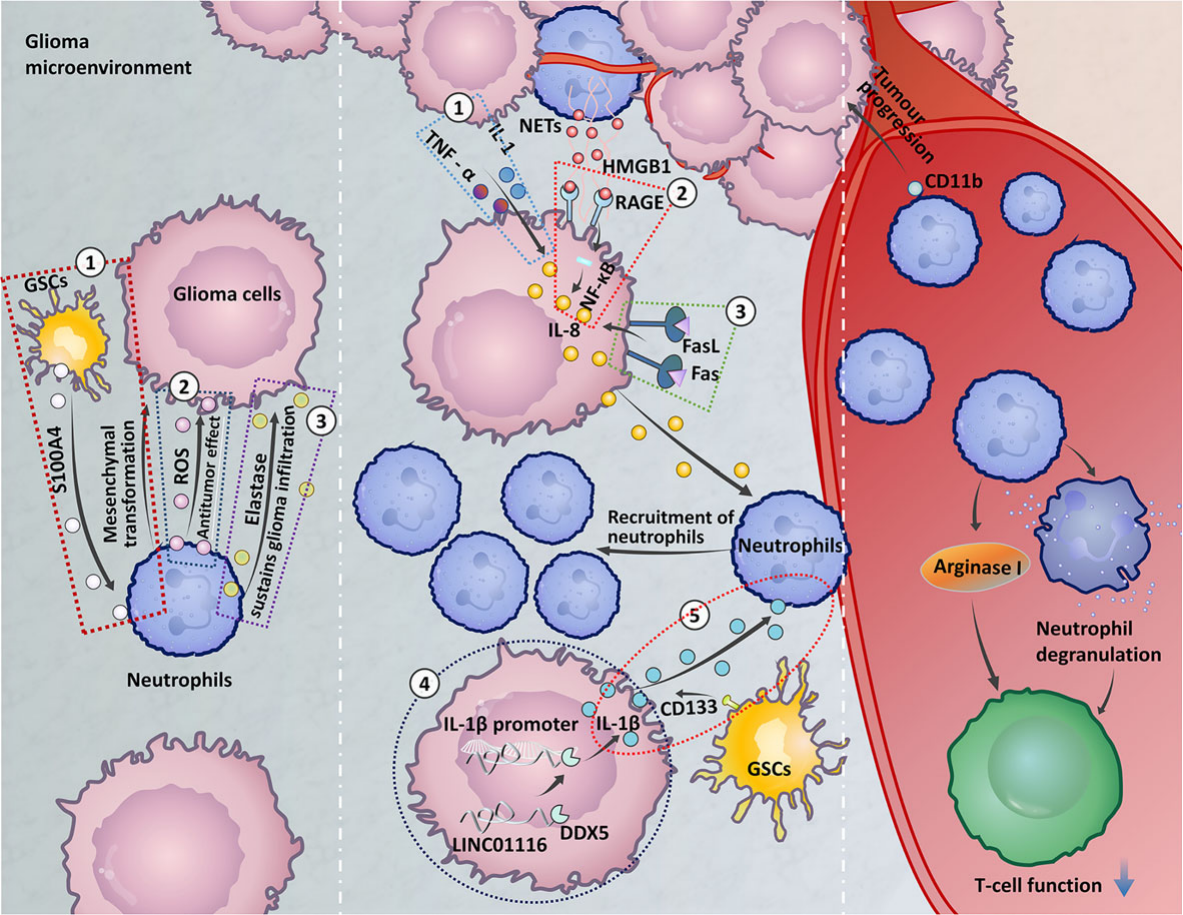
The role of circulating neutrophils and neutrophils at the tumour site. Left: ① Glioma stem cells (GSCs) express S100A4 to promote the mesenchymal transition of glioma cells (37). ② The release of reactive oxygen species (ROS) by neutrophils in the early stage of glioma development may be related to the antitumour neutrophil effect (89). ③ Neutrophils secrete elastase, which destroys brain tissue and aids glioma invasion (90). Middle: ① Astrocytoma and GBM cells express IL-1 and TNF and high levels of IL-8 under alpha stimulation, which recruit neutrophils (91). ② Neutrophils form high-mobility group box 1 (HMGB1) and bind to receptor for advanced glycation end products (RAGE) expressed in glioma tissues, activates the NF-κB signaling pathway to secrete IL-8, and promote neutrophil infiltration (92). ③ Expression of FasL on glioma cells activates Fas signaling in the TME to express IL-8, leading to neutrophil aggregation (93). ④ LINC01116 promotes the expression of IL-1β by recruiting the transcription regulator DDX5 to the IL-1β promoter, which promotes the recruitment of neutrophils (94). ⑤ The ectopic expression of CD133 induces an increase in IL-1β expression, which causes neutrophils to aggregate in the TME (95). Right: Neutrophil degranulation and elevated levels of serum arginase I induce immunosuppression in GBM patients (96). Neutrophil expression of CD11b is an early predictor of tumour progression (97).

The baseline neutrophil count is a current biomarker used to predict the efficacy of bevacizumab in the treatment of GBM (86). It has been found that an increased NLR has been associated with increased peritumoral infiltration of macrophages and upregulation of several cytokines, such as interleukin (IL)-6, IL-7, IL-8, IL-9, IL-12, IL-17, and IFNγ (87, 88). In the study of the immunosuppressive effect of GBM patient peripheral blood, it was found that peripheral cellular immunosuppression in GBM patients is correlated with increased neutrophil degranulation and elevated levels of serum arginase I (As shown on the right side of **Figure 1**) (98). Neutrophil degranulation is the process by which neutrophil cytoplasmic granules fuse with the cell membrane or phagosomal membrane, leading to the exocytosis of soluble granule proteins or exposure of membrane granule proteins to the cell surface (99). And arginase I is a factor known to be present within in granulocytes and has immunosuppressive activity (98). Arginase I expression suppress T cell function in patients with GBM and T cell function can be restored by targeting serum arginase I (96). Correlations of phenotypic characteristics between neutrophils in the blood and high-grade tumours have recently been reported. When compared to healthy controls, individuals with glioma expressed a few activation markers (CD11b, CD16, CD54, and CD63) and L-selectin (CD62L) at lower levels on neutrophils. Moreover, neutrophils showed higher expression of the surface receptor CD16 in the context of grade III gliomas in GBM (100). Activation of neutrophils expressing CD11b is an early predictor of tumour progression in GBM patients (97).

Taken together, these findings illustrate that circulating neutrophils play important roles in the diagnosis, OS, immunosuppression, tumour growth promotion, and treatment resistance of patients with glioma. Data demonstrate the association between elevated peripheral blood NLR levels and increased TANs infiltration (101). However, the molecular mechanisms by which the NLR is associated with TANs remain unknown.

## Different Immune Compositions in the Glioma Microenvironment

The normal brain has traditionally been recognized as an immune-privileged organ due to the presence of the endothelial blood-brain barrier (BBB) and the absence of a conventional lymphatic system (102). However, this viewpoint has recently been challenged, as even in the presence of an intact BBB, adaptive immune cells can traffic into the central nervous system (CNS) (103). Functional lymphatic vessels lining the dural sinuses were recently reported. These structures can carry immune cells from the cerebrospinal fluid and are connected to the cervical lymph nodes (104). Kipnis et al. described the cellular and molecular orchestration of the dural sinuses as a unique interface where the CNS and the immune system communicate with one another (105). Indeed, cells from the bone marrow, including neutrophils and monocytes, may directly from nearby bone marrow cavities in the skull and vertebrae (106–108). Moreover, in certain brain tumours, BBB dysfunction can be accompanied by increased leukocyte infiltration from the peripheral circulation (109). Nonetheless, the microenvironment of the glioma is generally immunosuppressive, with essentially no trafficking or patrolling by peripheral immune cells (110).

GBM cells produce cytokines, chemokines, growth factors and extra-cellular matrix modifying enzymes, extracellular vesicles and proteins to construct a favourable tumour microenvironment (111). Also, cells in TME interact with each other and with the neoplastic cells through different suppressor receptors like programmed cell death protein-1 (PD-1), T-lymphocyte-associated protein 4 (CTLA-4), CD70 and gangliosides that increase the tumour immune escaping (112, 113). The modulation of these cell populations in the brain TME could improve the efficacy of immunotherapy against brain malignancies.

In the case of glioma, the inflammation-enriched TME has many tumour-promoting effects (114). The glioma microenvironment exhibits a diverse immune cell landscape with substantial infiltration of resident microglia (115), circulating blood monocytes (macrophages) (116), dendritic cells (DCs), lymphoid cells, and neutrophils (117, 118). Microglia are tissue-resident macrophages that arise from embryonic yolk sac precursors (119). These cells regulate the innate immune response in the brain and play a major role in normal brain development (120–122). Few studies have investigated other populations of immune cells in the brain. Indeed, a recent discovery identified small populations of T cells and B cells that regulate microglial maturation and promote oligodendrocyte precursor cell proliferation, respectively (123, 124). TAMs consist of bone marrow-derived macrophages and tissue-resident microglia (125). In GBM, TAMs have a protumour role, and increased TAM accumulation is associated with increased tumour grade (126–129). There is increasing evidence that TAMs promote glioma growth and invasion (130). DCs are myeloid-derived cells that can stimulate T lymphocytes and natural killer (NK) cells to become potent antitumour effectors (131). Recent studies have reported the clinical effectiveness of DC-based vaccine therapy in malignant glioma (132). T cells, B cells, and NK cells migrate through the lymphatic system. Low levels of CD4^+^ T helper (Th) cells and CD8^+^ cytotoxic T lymphocytes (CTLs) within the T cell population have been shown to infiltrate gliomas (133). High levels of CD8^+^ CTLs are commonly regarded as having antitumoral activity, whereas high levels of CD4^+^ Th cells are related to favouring tumour development (134). NK cells are known to play a role in the apoptotic killing of both tumour cells and virus-infected cells (134). The role of B cells in glioma development is unclear. A comprehensive understanding of the complex glioma microenvironment will greatly expand the range of therapeutic strategies for this deadly disease.

Growing evidence has highlighted the role of neutrophils in promoting tumour progression in the brain TME. Neutrophil functions in the glioma microenvironment are described in more detail below. The modulation of neutrophils in the brain TME could improve the efficacy of therapy against brain malignancies.

## Neutrophil Recruitment

Neutrophils are generated under steady-state conditions from haematopoietic stem and progenitor cells in the bone marrow. However, during infection or cancer, neutrophils are used up in large quantities, and the steady-state condition is converted to emergency granulopoiesis (135). In mouse models and patients with invasive cancer, the spleen also produces neutrophils during cancer progression (136). Growth factors (G-CSF and granulocyte-macrophage colony-stimulating factor (GM-CSF)) and inflammatory cytokines (IL-6, IL-1β, and IL-17) produced by tumour cells, tumour-associated stromal cells, and tumour-infiltrating leukocytes (including T cells) can modulate haematopoiesis (48). G-CSF is the principal cytokine regulating neutrophil generation and differentiation (137, 138). In addition to G-CSF, stem cell factor, IL-6, and GM-CSF induce an increase in neutrophils (139–141). The chemokine receptors CXCR1 and CXCR2 are expressed by neutrophils, and activation of these receptors is key to neutrophil recruitment. Tumour-infiltrating leukocytes, endothelial cells, and fibroblasts express the CXC chemokine ligands CXCL1, CXCL2, CXCL5, CXCL6, and CXCL8 (also known as IL-8) (142, 143). The chemokine receptor CXCR2 was originally found to be expressed on neutrophils (144). In mouse colon cancer models, the chemokines CXCL1, CXCL2, and CXCL5 are CXCR2 ligands that are observed to promote neutrophil recruitment (143, 145–147). CXCR2 is also the receptor for IL-8 and mediates neutrophil activation. The expression of CXCR2 proteins in gliomas has been significantly correlated with glioma recurrence (148).

The mechanism of TANs recruitment to gliomas remains limited (In the middle of the **Figure 1**). IL-8 is associated with the recruitment of neutrophils *via* the activation of multiple intracellular signalling pathways (149). Glioma cells produce a cytokine-induced neutrophil chemoattractant, IL-8, which attracts granulocytes to the tumour site (150). Astrocytoma and GBM cells express high levels of IL-8 under stimulation with IL-1 and TNF-α, and IL-8 has chemotactic effects on human neutrophils (91). Neutrophils exert their functions through the formation of NETs. In glioma, high-mobility group box 1 (HMGB1) derived from NETs binds to receptor for advanced glycation end products (RAGE) expressed in glioma tissue, activating the NF-κB signalling pathway to promote IL-8 secretion, which promotes neutrophil infiltration (92). In addition, IL-1β is involved in many diseases and tissue inflammation (151). LINC01116, a long noncoding RNA expressed in glioma tissue, can promote IL-1β expression by recruiting the transcriptional regulator DDX5 to the IL-1β promoter. Then, IL-1β expression in glioma cells promotes TANs recruitment (94). CD133 is a surface marker of glioma stem cells (GSCs). Increases in the expression of IL-1β induced by ectopic expression of CD133 recruit neutrophils to the TME and increase neutrophil migration (95). FasL expression on gliomas activates Fas signalling in the TME, and glioma cells express IL-8 in response to Fas activation, which leads to an accumulation of neutrophils (93). In addition, a recent study reported the upregulation of CXCL8, ITGA3, and CXCL17 by brain metastases. These chemokines are involved in neutrophil tissue infiltration. Increased expression of MET was found in neutrophils in brain metastases, and MET has been related to the recruitment of immunosuppressive neutrophils. The increased expression of the cell-surface receptor CD117 was correlated with neutrophil migration and activation (152). Whether these chemokines are involved in neutrophil infiltration in the glioma microenvironment needs to be further investigated.

The TIMER2.0 database, R programming language, and so on have been used to analyse tyrosine protein tyrosine kinase binding protein, and CD96 expression has been correlated significantly with neutrophil infiltration (153, 154). Gene ontology (GO) enrichment analysis and gene set enrichment analysis (GSEA) showed that BLC7A was mainly enriched in neutrophil activation. Immunohistochemical (IHC) analysis revealed that low BCL7A expression was correlated with robust infiltration of neutrophils in gliomas (155). However, more studies are required to determine the underlying mechanisms.

## Neutrophils Acquire Unique Phenotypes in Glioma

Neutrophils have long been known to be responders in innate and adaptive immune responses that defend against infectious agents (156). Once neutrophils are recruited to the glioma microenvironment, they adopt new cellular and molecular identities.

IHC staining was used to detect neutrophil infiltration in human glioma tissues of different grades. The neutrophil infiltration level was positively correlated with glioma grade (36). In addition to the discovery of neutrophil cells infiltrating glioma tissue, in *in vitro* coculture models, neutrophils may be partially responsible for enhanced glioma proliferation (Summarized on the left side of **Figure 1**) (37). Subsequent studies investigating neutrophil function in depth described that neutrophils secrete elastase. Neutrophils elastase is a neutral protease and cytotoxic mediator that can damage brain tissue and aid in glioma invasion (90). Apart from invasion, neutrophils modulate tumour angiogenesis. S100A4 is a novel biomarker expressed in GSCs (157) that induces the tumorigenic activity of neutrophils. Neutrophils promote the mesenchymal transformation of gliomas *via* increased expression of S100A4 within the gliomas and increase vascularization, which induces resistance to anti-VEGF therapy (37). In mouse tumours, PMN-MDSCs and TANs express Ly6G (158, 159). Radiation-induced infiltrating Ly6G^+^ neutrophils secrete Nitric oxide (NO) that promotes the activity of the NOS-ID4 signalling axis, which converts GBM cells into GSCs, this conversion is negatively associated with survival and radiation therapy outcomes (160). It is important to note that telomerase reverse transcriptase mutation is accompanied by neutrophil infiltration and neutrophil chemokine expression in the IDH-wt glioma microenvironment, which may be partly responsible for the poor prognosis of IDH-wt gliomas (161). Furthermore, the reduced neutrophil infiltration in IDH-mt gliomas may contribute, in part, to the improved clinical outcomes observed in these patients (162).

Although previous studies have shown that neutrophils contribute to the malignant progression of gliomas, neutrophils can also limit glioma growth. It was recently reported that neutrophils are recruited during the early stages of glioma development and exert an antitumour function in tumour-bearing mice. Increased reactive oxygen species (ROS) release levels might be responsible for the role of antineoplastic neutrophils. Unfortunately, as the tumour progressed, neutrophils lost the ability to prevent tumour progression (89). The antitumorigenic property of neutrophils during early stages of glioma suggests that these cells may contribute to improved immunotherapeutic outcomes in patients with glioma.

## Effect of Neutrophils on T Cells

**Figure 2:**
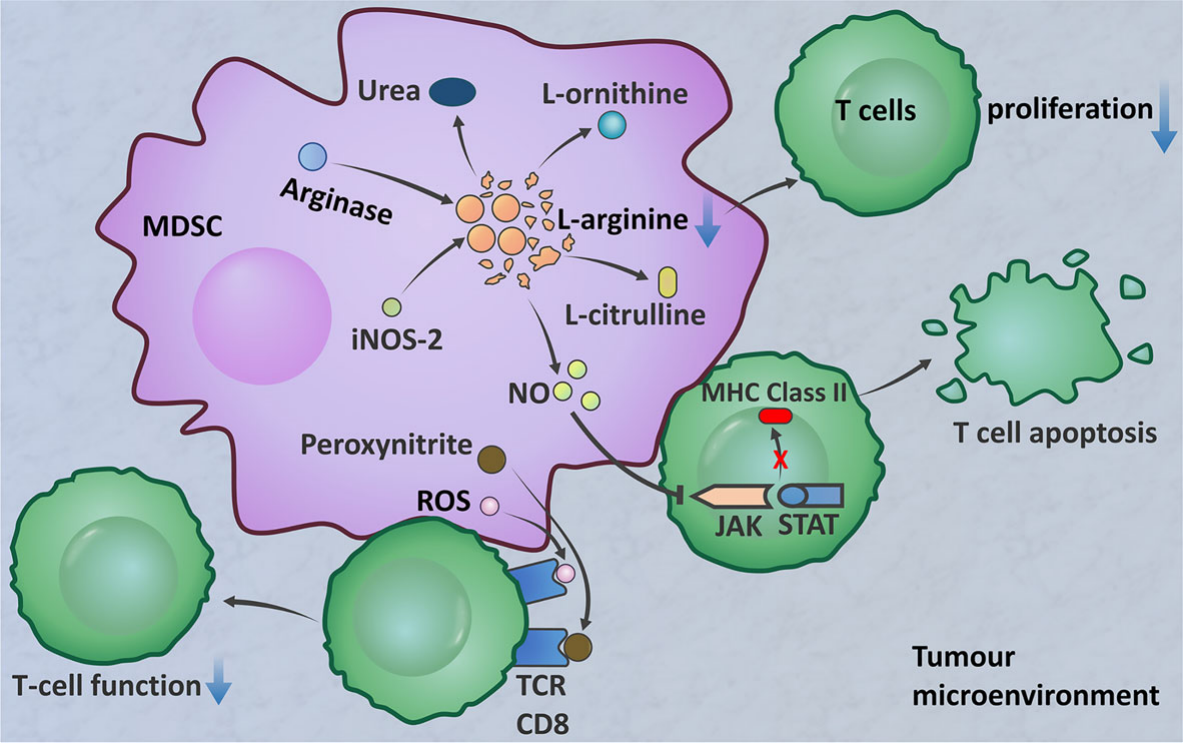
The immunosuppressive function of MDSCs. MDSCs produce arginase, which decomposes l-arginine into urea and l-ornithine (163, 164). MDSCs express iNOS2, which converts l-arginine into NO and l-citrulline (165). L-Arginine deficiency inhibits T cell proliferation. NO interferes with JAK/STAT signalling proteins, inhibits the transcription of MHC class II genes, and induces T cell apoptosis (166–168). MDSCs produce high levels of peroxynitrite and ROS when in direct contact with T cells to induce TCR and CD8 molecular modification, resulting in CD8^+^ T cells losing the ability to bind to the pMHC complex; this results in nonresponse of peripheral blood tumour-specific CD8^+^ T cells (169).

Based on MDSC function, PMN-MDSCs should refer to neutrophil subsets with proven immunosuppressive activity. MDSCs in the tumour site play a major role in T cell suppression (The immunosuppressive function of MDSCs is summarized in **Figure 2**). The important factors implicated in the MDSC-mediated suppression of T cell function include the metabolism of L-arginine, increased production of ROS, and increased levels of peroxynitrite (ONOO^−^) (170). M-MDSCs and PMN-MDSCs regulate different aspects of immune suppression. M-MDSCs suppress the T cell response by utilizing NO, whereas PMN-MDSCs use ROS, peroxynitrite, and arginase to mediate immune suppression (170, 171). Because of the increased arginase activity of MDSCs, L-arginine is catabolized into urea and L-ornithine. The created L-arginine deficiency inhibits T cell proliferation (163, 164). MDSCs are induced to express inducible nitric oxide synthase-2 (iNOS2), which converts L-arginine into NO and L-citrulline (165). NO is thought to interfere with T cell JAK/STAT signalling proteins required for T cell activation, inhibit MHC class II gene transcription, and induce T cell apoptosis (166–168). ROS are another important factor that mediate the immunosuppressive activity of MDSCs, which has been demonstrated in *in vitro* studies (172–174). MDSCs produce high levels of peroxynitrite and ROS when in direct contact with T cells. The superoxide anion ( $O_{2}^{-}$ ) interacts with NO to form peroxynitrite. An *in vivo* experimental model found that MDSCs produce ROS and peroxynitrite to induce modification of TCR and CD8 molecules, resulting in CD8^+^ T cells losing the ability to bind to pMHC complexes and inducing nonresponsiveness in tumour-specific CD8^+^ T cells in the peripheral blood (169). Comparison of MDSCs between the peripheral blood and TME shows that tumour MDSCs have more effective inhibitory activity. After migrating to a tumour, MDSCs are exposed to inflammation and hypoxia in the TME. This results in increases in arginase and iNOS, downregulation of ROS production, and upregulation of inhibitory PD-1 ligand (PD-L1) expression on the MDSC surface (171).

In gliomas, the abilities of MDSC subsets to express arginase I and produce ROS have been confirmed. Arginase I is expressed in tumour-derived MDSCs, predominantly M-MDSCs. Only a small portion of MDSCs in the blood of glioma patients express arginase. In contrast, both MDSC subsets can produce ROS (175). MDSCs were found to be increased in the peripheral blood of patients with GBM, and the largest population, comprising more than 60% of cells, was neutrophil MDSC subsets. MDSCs in the peripheral blood of patients with GBM were shown to suppress T cell IFN–γ production (176). Immunohistochemistry confirmed that CD15^+^ granulocytic MDSC (PMN-MDSC) subsets are dominant in glioma tissue (177). Blood-derived neutrophilic MDSCs inhibit T cell proliferation *in vitro*. There is a correlation between granulocytic MDSCs and effector memory CD4^+^ T cells in gliomas. Effector memory CD4^+^ T cells are dysfunctional and express high levels of PD-L1, an immunoinhibitory receptor that is involved in functional T cell exhaustion (175). The results of these studies have important clinical implications for immune-based interventions in GBM. Strategies to target MDSCs in peripheral blood and tumour tissue should be implemented into immunotherapeutic approaches.

## Potential Application of Neutrophils in Glioma Therapy

The treatment of gliomas has been particularly challenging due to the high invasive growth and treatment resistance of these tumours (178, 179). In the context of glioma, neutrophils typically promote cancer cell proliferation, immunosuppression, and angiogenesis in support of tumour growth and metastasis (9, 76). Hence, significant attention has been drawn towards development of glioma immunotherapies targeting these neutrophils; either depleting them from tumour, blocking their infiltration, or using neutrophil-delivered drug system to exert immunostimulatory/tumoricidal properties (180–184).

### Inhibiting Neutrophils

Blocking VEGF to inhibit neovascularization has emerged as a primary strategy for glioma treatment (37, 185). Bevacizumab is a humanized monoclonal antibody against VEGF that improves progression-free survival in GBM patients (186). However, neutrophil infiltration into tumours is significantly correlated with acquired resistance to anti-VEGF therapy (37). Therefore, further research is needed to determine the exact mechanism by which neutrophils mediate anti-VEGF treatment resistance in GBM and to propose potential approaches for glioma treatment.

In addition, as mentioned above, in patients with glioma, increased neutrophil infiltration is associated with glioma progression and a poor prognosis. R. E. Kast et al. hypothesized that dapsone, an antibiotic, could target neutrophils by blocking IL-8-mediated neutrophil infiltration and subsequently limiting glioma cell migration (182). The results demonstrated in a modified rat T9 GBM model that glioma cells genetically engineered to secrete IL-6 invoke an effective, antitumour response in which the early stages may be mediated by neutrophils (181). These studies provided valuable information on neutrophils response to glioma *in vitro* and *in vivo*. In contrast to previous neutrophil depletion approaches, Yun Chang et al. established a new platform for producing neutrophils. They used chimeric antigen receptors (CARs) to enhance neutrophil antitumour cytotoxicity for targeted therapy of glioma (180). This strategy may complement current standard glioma treatments and boost their efficacy. Other strategies of cancer immunotherapy are to prevent the interaction between PD-1 on T cells and PD-L1 on tumour cells or host cells. Anti-neutrophil reagents have been observed to enhance the treatment efficacy of PD-1 inhibitors in most glioma mouse models (187) (**Table 2**). Future investigation is encouraged to target neutrophils in gliomas to alleviate their negative effects on PD-1 inhibitors.

**Table 2:** Studies to treat glioma by targeting neutrophils.

Neutrophil-targeted agent	Target	Test Systems	References
Dapsone	IL−8	*In vitro* human	(182)
anti-Ly6G antibody	Neutrophils	*In vivo* mouse	(187)
IL-6	Recruit antitumour neutrophils	*In vivo* rat	(181)
Neutrophils	Antitumour neutrophils	*In vitro* human and *in vivo* mouse	(180)

### Therapeutic Targeting of Brain TME by Neutrophils

**Figure 3:**
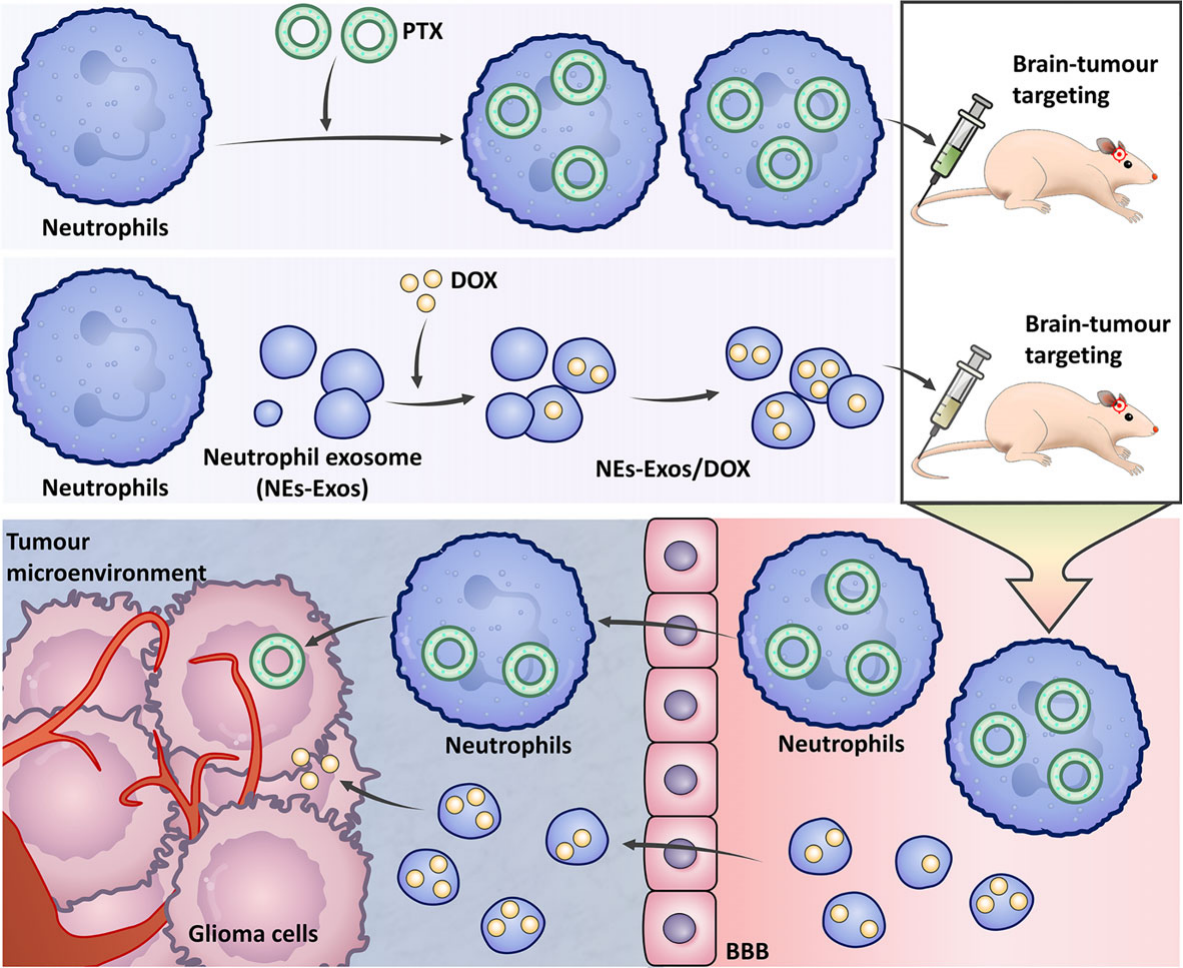
The strategy uses neutrophils to treat gliomas. Neutrophils carry paclitaxel (PTX) liposomes to treat gliomas. In the neutrophil-derived exosome (NEs-Exos) drug delivery system, the anticancer drug doxorubicin (DOX) is loaded into the nanocarrier for the treatment of gliomas.

Drug delivery directly into the CNS is a strong strategy because it circumvents the obstacle of the BBB (These methods are summarized in **Figure 3**) (183, 184). Neutrophils have the natural abilities to penetrate glioma sites and cross the BBB. Treatment with neutrophils carrying paclitaxel (PTX)-loaded liposomes produced superior suppressive effects on tumour recurrence in glioma mouse models (184). A neutrophil-derived exosome (NEs-Exos) drug delivery system for the treatment of glioma was recently reported. The anticancer drug doxorubicin (DOX) was loaded into this nanocarrier, which could efficiently cross the BBB into the brain and target inflamed brain tumours. NEs-Exos have been confirmed to efficiently suppress tumour growth and prolong survival time (183). These novel strategies hold positive clinical prospects for brain targeting if explored further in the right direction.

### Inhibiting the Formation of NETs

The presence of NETs in tumours of CNS has rarely been reported. Recently, NETs were detected in grade IV glioma tissues by staining for MPO and CitH3. The levels of NETs in high-grade glioma tissues were significantly higher than those in low-grade glioma tissues. Furthermore, NETs participate in the proliferation and invasion of GBM cells by binding HMGB1 to RAGE to activate the NF-κB signaling pathway (92).

Injection of DNase I into experimental animals degraded extracellular DNA fibres and significantly inhibited the invasion and metastasis of pancreatic cancer cells (188). A study by Meurer et al. reported host DNase 1 promoted the killing of S. suis by neutrophils by cleaving DNA fibers in NETs (189). In addition to DNase, certain drugs or compounds have been shown to inhibit or destroy NETs and may play a therapeutic role in CNS diseases. Cl-amidine and BB-Cl-amidine are nonspecific PAD inhibitors that inhibit PAD4 and reduce the formation of NETs (190). HMGB1 plays an important role in ischaemic cerebral infarction and promotes the production of NETs. Studies have shown that the use of anti-HMGB1 antibodies can reduce the formation of NETs (191). The antidiabetic drug metformin has also been shown to reduce NETs concentrations *in vitro* (192). These drugs targeting NETs may arouse interest in treating gliomas. Futures potential therapeutic strategy for gliomas are needed to refine our knowledge on NETs.

## Discussion

The important role of neutrophils in tumour progression and their potential as therapeutic targets have been extensively studied in recent years (182, 193). To date, studies on neutrophils in cancer have investigated not only the ability of these cells to promote or prevent tumour progression but also the recruitment mechanism of neutrophils and their phenotypic classification (41). Each of these findings opens up new opportunities for therapeutic intervention in glioma patients.

The presence and significance of neutrophils in gliomas have long been overlooked. Clarifying the roles of neutrophils in the peripheral blood and TME of patients with glioma will help improve the potential of targeted glioma therapies and incorporate these cells into current treatment regimens. Circulating neutrophils are closely correlated with clinicopathological parameters such as tumour stage, tumour progression, and OS, so neutrophils can be used as biomarkers for diagnosis and prognosis (194–196). Most previous studies in patients with glioma have shown that neutrophils infiltration at the tumour site has negative effects on tumour progression, patient survival, and treatment response (197–199). Further study of the effects of neutrophils in the TME and analysis of their diversity has revealed new insights into TANs in gliomas, showing that neutrophils can directly exert important antineoplastic activity (89, 200). The goal of the previous hypothetical approach was to block neutrophils from infiltrating into the tumour site (201), and the discovery of the role of antitumour neutrophils provides a new way to improve the efficiency of current treatments (89). In conclusion, neutrophils perform different functional roles in the progression of glioma. Targeting neutrophils can block the growth of glioma cells and improve the immune response in the lesional area, and tumour progression can also be systematically inhibited using targeted metabolic drug delivery systems based on neutrophils (182–184). In addition, many drugs or compounds have been shown to inhibit the formation of NETs through different mechanisms (202, 203). We speculate that the use of these drugs or compounds is beneficial for the treatment of gliomas and hope to confirm this in future studies.

This is expected to be a new direction for the clinical treatment of glioma. However, the role of neutrophils in gliomas has not been sufficiently studied, and more studies are needed to elucidate the role and mechanism of neutrophils in gliomas. In addition, the clinical application prospects of neutrophils, whether for neutrophil recruitment or NETs, are expected to be confirmed in subsequent studies. Therefore, we hope that this paper can provide inspiration or useful information for follow-up study on neutrophils in glioma to promote progress in the diagnosis and treatment of glioma.

## Author Contributions

GW wrote the manuscript; JW retrieved literature; PW, YZ, and CN critically revised the manuscript. All authors have read and approved the final manuscript.

## Funding

This research was supported by the Anhui Province Key Laboratory of Translational Cancer Research (Bengbu Medical College) (KFZZ202203).

## Conflict of Interest

The authors declare that the research was conducted in the absence of any commercial or financial relationships that could be construed as a potential conflict of interest.

## Publisher’s Note

All claims expressed in this article are solely those of the authors and do not necessarily represent those of their affiliated organizations, or those of the publisher, the editors and the reviewers. Any product that may be evaluated in this article, or claim that may be made by its manufacturer, is not guaranteed or endorsed by the publisher.
